# Lack of collagen α6(IV) chain in mice does not cause severe-to-profound hearing loss or cochlear malformation, a distinct phenotype from nonsyndromic hearing loss with *COL4A6* missense mutation

**DOI:** 10.1371/journal.pone.0249909

**Published:** 2021-04-13

**Authors:** Shaoying Tang, Tomoko Yonezawa, Yukihide Maeda, Mitsuaki Ono, Takahiro Maeba, Toru Miyoshi, Ryusuke Momota, Yasuko Tomono, Toshitaka Oohashi

**Affiliations:** 1 Department of Molecular Biology and Biochemistry, Okayama University Graduate School of Medicine, Dentistry and Pharmaceutical Sciences, Okayama, Japan; 2 Department of Otolaryngology-Head and Neck Surgery, Okayama University Graduate School of Medicine, Dentistry and Pharmaceutical Sciences, Okayama, Japan; 3 Department of Cardiovascular Medicine, Okayama University Graduate School of Medicine, Dentistry and Pharmaceutical Sciences, Okayama, Japan; 4 Department of Human Morphology, Okayama University Graduate School of Medicine, Dentistry and Pharmaceutical Sciences, Okayama, Japan; 5 Division of Molecular and Cell Biology, Shigei Medical Research Institute, Okayama, Japan; University of Washington, UNITED STATES

## Abstract

Congenital hearing loss affects 1 in every 1000 births, with genetic mutations contributing to more than 50% of all cases. X-linked nonsyndromic hereditary hearing loss is associated with six loci (DFNX1-6) and five genes. Recently, the missense mutation (c.1771G>A, p.Gly591Ser) in *COL4A6*, encoding the basement membrane (BM) collagen α6(IV) chain, was shown to be associated with X-linked congenital nonsyndromic hearing loss with cochlear malformation. However, the mechanism by which the *COL4A6* mutation impacts hereditary hearing loss has not yet been elucidated. Herein, we investigated *Col4a6* knockout (KO) effects on hearing function and cochlear formation in mice. Immunohistochemistry showed that the collagen α6(IV) chain was distributed throughout the mouse cochlea within subepithelial BMs underlying the interdental cells, inner sulcus cells, basilar membrane, outer sulcus cells, root cells, Reissner’s membrane, and perivascular BMs in the spiral limbus, spiral ligament, and stria vascularis. However, the click-evoked auditory brainstem response analysis did not show significant changes in the hearing threshold of *Col4a6* KO mice compared with wild-type (WT) mice with the same genetic background. In addition, the cochlear structures of *Col4a6* KO mice did not exhibit morphological alterations, according to the results of high-resolution micro-computed tomography and histology. Hence, loss of *Col4a6* gene expression in mice showed normal click ABR thresholds and normal cochlear formation, which differs from humans with the *COL4A6* missense mutation c.1771G>A, p.Gly591Ser. Therefore, the deleterious effects in the auditory system caused by the missense mutation in *COL4A6* are likely due to the dominant-negative effects of the α6(IV) chain and/or α5α6α5(IV) heterotrimer with an aberrant structure that would not occur in cases with loss of gene expression.

## Introduction

The ear, a precise organ composed of the external, middle, and inner ear, that is heavily involved in the auditory system and balance. Auditory function plays an important role in communication and learning abilities [1]. The process of hearing begins when sound is conducted by air or bone. During air conduction, sound waves are collected, causing vibration of the tympanic membrane and ossicular chain. Sound waves also transfer through the skull, which is referred to as bone conduction. Vibrations within the endolymph through stapedial vibrations stimulate auditory receptors in the cochlea.

**Fig 1:**
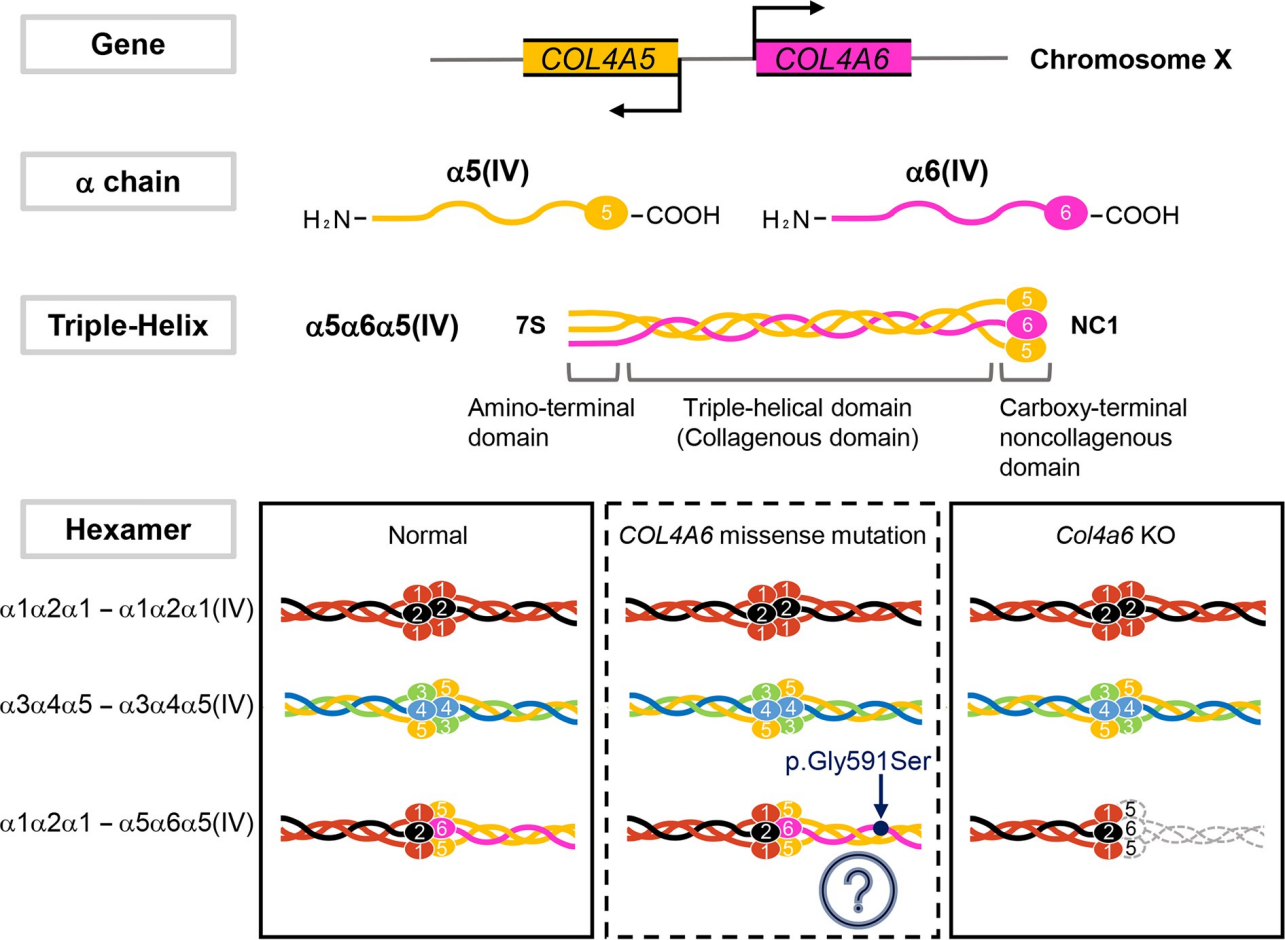
Schematic view of the gene structure and assembly of collagen IV. The *COL4A6/Col4a6* gene is located on the X chromosome and paired with *COL4A5/Col4a5* in a head-to-head manner. *COL4A1/Col4a1* and *COL4A2/Col4a2*, and *COL4A3/Col4a3* and *COL4A4/Col4a4* pairs are located in a head-to-head manner on their respective chromosomes (not shown). Each chain comprises a 400 nm long triple-helical domain, which contains many interruptions in the Gly-X-Y repeated sequence. The globular noncollagenous domain (NC1) is located at the C-terminal end, while the 7S domain is at the N-terminal end. Three α chains assemble the triple-helical molecule (triple-helix). The heterotrimers extracellularly form a hexamer. α1α2α1(IV) and α5α6α5(IV) bind to each other through the NC1 domain [α1α2α1-α5α6α5(IV)] and the 7S domains (not shown), which facilitates their assembly to a higher-ordered supra structure. α1α2α1(IV) is widely distributed, whereas α3α4α5(IV) and α5α6α5(IV) distribution is tissue specific [7–9, 12]. In *Col4a6* KO mice, the loss of α6(IV) chain expression inhibits the formation of the α5α6α5(IV) heterotrimer. This study showed that the phenotypes of the auditory system in *Col4a6* KO mice were distinct from those of individuals with the *COL4A6* missense mutation c.1771G>A, p.Gly591Ser. The presence of the mutant protein α6(IV) chain and/or α5α6α5(IV) may induce deleterious effects on the cochlea. The pathogenesis involved in congenital hearing loss warrants further investigations.

The cochlea, comprising the modiolus and osseous cochlear duct, is a spiral-shaped cavity in the bony labyrinth. The membranous cochlear duct is located in the osseous cochlear duct and includes the spiral limbus, basilar membrane, organ of Corti, and stria vascularis in the spiral ligament, which contribute to the mechanical-electrical signal conversion and ion transportation during sound conduction [1, 2]. The extracellular matrix (ECM) also contributes to the auditory system; collagen and cochlin are the most abundant ECM components in the cochlea. Fibrillar collagen primarily provides tissue stability and strength, while cochlin interacts with collagen molecules [3]. Interestingly, a recent study has shown that cochlin also plays a role in innate immune responses within the inner ear [4]. Histologically, basement membranes (BMs) are present in the membranous labyrinth of the cochlea. The BM is a cell-adherent and sheet-like ECM found beneath the epithelium and endothelium, and surrounding smooth muscle cells, and adipocytes. The primary role of BMs is to provide tissue structure, divide the tissue into compartments, and influence cell behavior [5]. Collagen IV, laminin, perlecan, and nidogen are major components of BMs. Collagen IV comprises six genetically distinct α chains from α1(IV) to α6(IV) encoded by *COL4A1* to *COL4A6*. Three unique triple-helical forms have been identified: α1α2α1, α3α4α5, and α5α6α5 (Fig 1). Furthermore, these protomers are extracellularly assembled into three hexamers, namely, α1α2α1-α1α2α1, α1α2α1-α5α6α5, and α3α4α5-α3α4α5 [6–8]. Previous reports showed that α1(IV) and α2(IV) chains are abundant in all BMs, whereas α3(IV) to α6(IV) chains have a tissue-specific distribution. It is believed that the differential molecular composition of BM contributes to its specific biological roles in tissues. Specifically, the α6(IV) chain is preferentially found in subepithelial- and smooth muscle cell-BMs in various organs [9–15].

Various genes encoding ECM or ECM-related proteins reportedly cause hereditary hearing loss, including *COL4A6*, which encodes the sixth alpha chain of collagen IV [16, 17]. Previously, we established *Col4a6* knockout (KO) mice, which are apparently healthy and fertile, but show a developmental delay in keratinization of the oral mucosal epithelium [18–20]. In zebrafish, *col4a5* and *col4a6* are essential for BM integrity, supporting the axogenesis of granule cells and retinal ganglion cells [21]. Thus, the collagen α6(IV) chain is a crucial element of BMs; however, its physiological role remains elusive.

Recently, the missense mutation (c.1771G>A, p.Gly591Ser) in *COL4A6* was reported to be associated with X-linked congenital nonsyndromic hearing loss with cochlear malformation (OMIM: #300914); indeed, all the male subjects in this family experienced severe-to-profound hearing loss at all frequencies tested (0.125 to 8 kHz). Bioinformatic analysis predicted that the p.Gly591Ser missense mutation reduces the triple-helical conformational stability and triggers quaternary structure disassembly [22]. However, the impact of this mutation in the collagen α6(IV) chain *in vivo* remains unclear.

In this study, we investigate the collagen α6(IV) chain distribution in mouse cochlea and its effects on the cochlear formation and auditory function using a *Col4a6* KO mouse model. Overall, our study demonstrated the detailed distribution of collagen α6(IV) chain in mouse cochlea and showed that loss of expression of *Col4a6* in mice does not cause abnormalities in the cochlear structure or severe-to-profound hearing loss, which occurs in humans with missense mutation (c.1771G>A, p.Gly591Ser) of *COL4A6*.

## Materials and methods

### Animals

Male *Col4a6* KO and WT mice (8-week-old) were used in this study. *Col4a6* KO mice were generated by replacing part of exon 2 and intron 2 with a neomycin cassette in the *Col4a6* gene in the 129 SV/J background, as previously described, and subsequently backcrossed with C57BL/6J (Charles River) over ten generations [18, 19].

This study was conducted in strict accordance with the Policy on the Care and Use of Laboratory Animals, Okayama University. The protocol was approved by the Animal Care and Use Committee of the Okayama University (Protocol Number: OKU-2020035).

### Immunohistochemistry

The temporal bone was dissected from the mice (8-week-old) anesthetized with intraperitoneal xylazine (8 mg/kg) and ketamine (80 mg/kg), and snap-frozen in super Cryoembedding medium (SECTION-LAB Co. Ltd., Hiroshima, Japan). Then, 6 μm serial cryosections were prepared using Kawamoto’s film methods, as previously described with some modifications [20]. Briefly, the sections were fixed with acetone for 20 min followed by treatment with 6 M urea in 0.1 M glycine-HCl buffer (pH 3.5) to expose the epitope. Subsequently, 1% bovine serum albumin in phosphate buffered saline was used to block non-specific binding of antibodies for 1 h. The following rat monoclonal antibodies were used: H11 (1:100); H22 (1:100); fluorescein isothiocyanate labeled-M26 (1:10); 129 (1:100); b42 (1:100); H53 (1:300); and B66 (1:10), recognizing collagen α1(IV), α2(IV), α2(IV), α3(IV), α4(IV), α5(IV), and α6(IV) chains (generated by Dr. Y Tomono and Y Sado in Shigei Medical Research Institute, Okayama, Japan) [9, 11, 23]. Antibodies against CD31 (1:50, ab28364; Abcam, UK), perlecan (1:1,000, A7L6; Millipore, Burlington, MA, USA), laminin α1 (1:200, AL-1; Chemicon, Temecula, CA, USA), laminin α2 (1:1,000, 4H8-2; Sigma, Saint Louis, MO, USA), laminin γ1 (1:2,000, A5; Chemicon), and nidogen-1 (1:2,000; kindly gift by Dr. Takako Sasaki, Oita University) were used [24]. For the secondary antibody, Alexa Fluor® 488-conjugated goat anti-rat IgG (1:1,500; Invitrogen, Carlsbad, CA, USA), Alexa Fluor® 594-conjugated goat anti-rat IgG (1:1,500; Invitrogen), and Alexa Fluor® 594-conjugated donkey anti-rabbit IgG (1:1,500; Invitrogen) were used. Nuclei were stained with Hoechst 33258 (2 μM; Polysciences, Warrington, PA, USA). All images were obtained with a KEYENCE BZ-X710 microscope (Keyence, Osaka, Japan), and processed using BZ-X Analyzer software (Keyence). The experiment was repeated using three WT and three *Col4a6* KO mice.

### Auditory function

Auditory function was assessed by click-evoked auditory brainstem response (ABR) as previously described with minor modifications [25, 26]. *Col4a6* KO mice (n = 5) and WT mice (n = 5) were anesthetized with intraperitoneal xylazine (8 mg/kg) and ketamine (80 mg/kg). The generation of acoustic stimuli and simultaneous recording of evoked potentials were performed using Tucker Davis Technologies (TDT) ABR system (RA16, PA5, RP2.1, TDT, Gainesville, FL, USA). ABRs were evoked with clicks and the sound stimuli were applied in 5-dB SPL steps from 90 to 0 dB SPL. The click-ABR threshold was determined as the minimum sound pressure level at which the click-ABR waveform was visible on the recording monitor.

### Statistical analysis

Statistical analysis was performed using Student’s unpaired *t*-test. The data are presented as the mean ± standard deviation (SD). *P* < 0.05 was considered statistically significant.

### Micro-tomographic analysis (micro-CT)

The temporal bone was dissected from *Col4a6* KO and WT mice (8-week-old) anesthetized with intraperitoneal xylazine (8 mg/kg) and ketamine (80 mg/kg) and fixed with 10% formalin in neutral buffer at 4°C for 24 h. The samples were analyzed by micro-CT (SkyScan 1174 compact micro-CT, Bruker, Belgium) as described previously with some modifications [27]. Scans were performed at a resolution of 6.5 μm, after which 1,004 sections were reconstructed to produce the 3-D image of the petrous portion using SkyScan software (NRecon, CTAn, CTvol, and CTvox, SkyScan). In addition, to compare the shape and size of the cochlea between WT and *Col4a6* KO mice, DataViewer software was used according to the manufacturer’s protocol (Bruker). Briefly, we selected the volume of interest (VOI) in a 2-dimensional (2-D) plane with a clear view of the cochlea using WT scan data as a reference. The software automatically generated three orthogonal views of the best-matched 2-D image from the scan data of *Col4a6* KO as a target to the reference image. In the 2-D overlay image of WT and *Col4a6* KO, the matching degree was automatically represented by a color map based on the intensity of the radiography using DataViewer. The analysis was repeated using six cochleae from three mice of each genotype (WT, n = 3; KO, n = 3).

### Hematoxylin and eosin (HE) staining

The temporal bone was dissected from *Col4a6* KO and WT mice (8-week-old) anesthetized with intraperitoneal xylazine (8 mg/kg) and ketamine (80 mg/kg) and fixed in 4% paraformaldehyde in 0.1 M phosphate buffer (pH 7.2). After decalcification with 0.5 M EDTA (pH 7.5; Wako, Japan) for 7 days at room temperature, the cochleae were dehydrated and embedded in paraffin and 5 μm serial sections were prepared using a microtome (MICROM, HM 335E, Leica, Wetzlar, Germany). A new hematoxylin solution (Muto Pure Chemicals, Japan) and pure eosin solution (Muto Pure Chemicals) were used. All images were obtained with a KEYENCE BZ-X710 microscope (Keyence) and processed using BZ-X Analyzer software (Keyence). The experiment was repeated using three WT and three *Col4a6* KO mice.

## Results

### Distribution of collagen α6(IV) chain in the mouse cochlea

**Fig 2:**
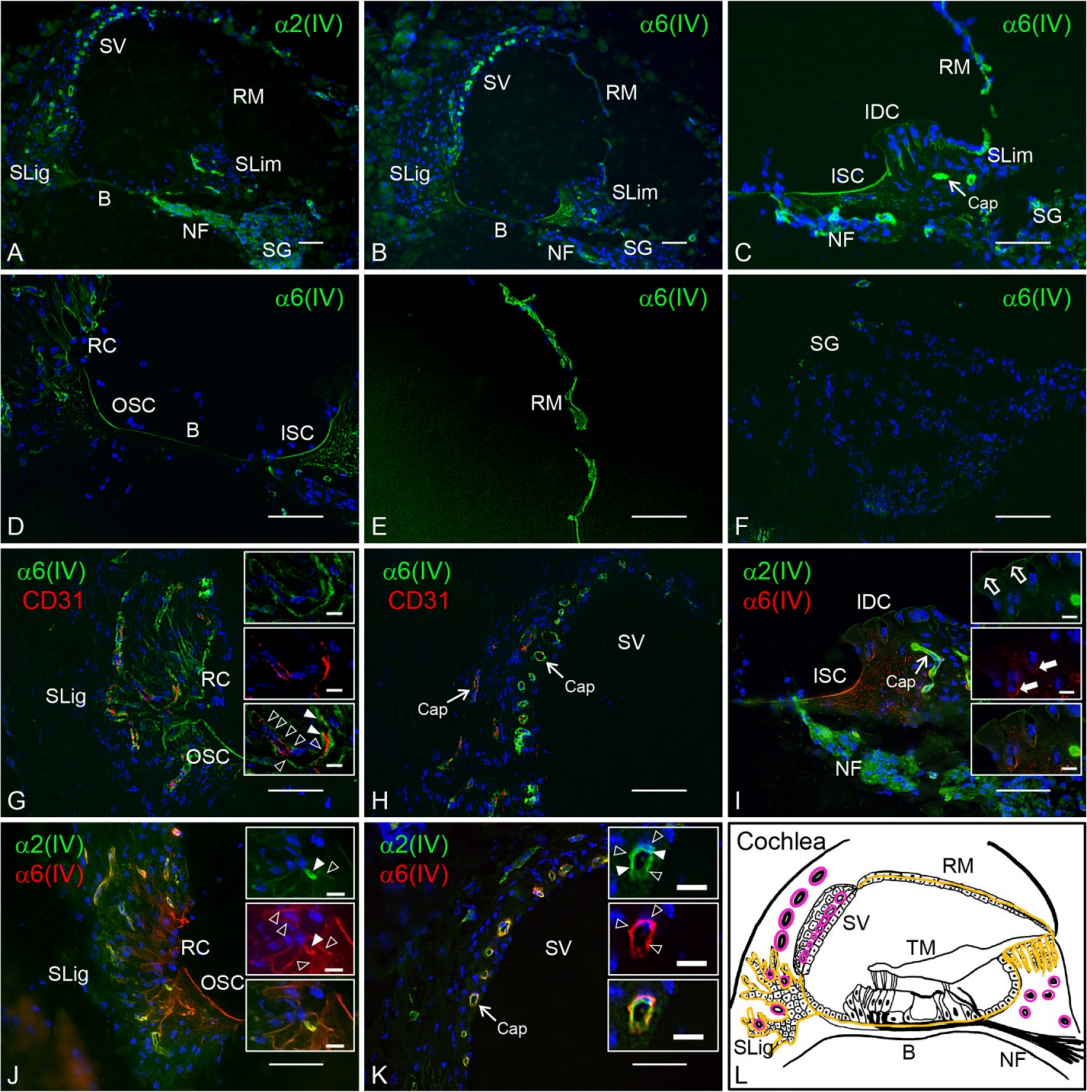
Distribution of collagen α6(IV) chain in the adult mouse cochlea. Sections were immunostained using antibodies against collagen α2(IV) (A, I–K: green) and α6(IV) (B–H: green; I–K: red) chains, and CD31 (G, H: red) for the detection of endothelial cells. Subepithelial BMs and perivascular BMs are indicated by open arrowheads and solid arrowheads in the insets, respectively (G, J, K). (I) The superficial plate and invaginated region of the interdental cells network are indicated by open arrows and solid arrows in insets, respectively. (L) Schematic diagram of the distribution of collagen α6(IV) chain [yellow line: subepithelial BMs; pink line: perivascular BMs]. The negative control was established by immunostaining with only the secondary antibody and no specific reaction was observed (S2 Fig). Nuclei were counterstained with Hoechst 33258 (blue). SLim: spiral limbus; B, basilar membrane; SLig, spiral ligament; SV, stria vascularis; RM, Reissner’s membrane; NF, neural fasciculus; SG, spiral ganglion; IDC, interdental cells; ISC, inner sulcus cells; OSC, outer sulcus cells; RC, root cells; Cap, capillary. Scale bars = 50 μm. Scale bars in inset = 10 μm.

To examine the distribution of the collagen α6(IV) chain in the mouse cochlea, we performed immunohistochemistry. Several BMs were found in the cochlea, including the subepithelial BMs of interdental cells, inner sulcus cells, basilar membrane, outer sulcus cells, root cells, and Reissner’s membrane; perivascular BMs in the spiral limbus, spiral ligament, and stria vascularis; and perineural BMs in the nerve fasciculus and spiral ganglion [28, 29]. We confirmed that both collagen α1(IV) and α2(IV) chains were present in all BMs in the mouse cochlea, as described previously (Fig 2A and S1 Fig) [30]. Results show that the collagen α6(IV) chain was observed in the subepithelial (Fig 2B–2E) and perivascular BMs (Fig 2B, 2D and 2G–2I), but not in the perineural BMs (Fig 2B and 2F).

For further characterization, we compared the localization of the collagen α6(IV) chain with that of the collagen α2(IV) chain using immunohistochemistry. The interdental cells are oval or round in shape and connect to form a comb-shaped network. The surface of the spiral limbus beneath the tectorial membrane is covered by flat processes of the interdental cells [31]. Although both the α2(IV) and α6(IV) chains were detected as comb-shaped networks, the immunoreactivity of α6(IV) chain was higher in the invaginated region than in the superficial plate of the network (Fig 2I). In the spiral ligament, the α6(IV) chain appeared conspicuously in the radiated epithelial BMs surrounding the root cells and beneath the outer sulcus cells compared with the α2(IV) chain (Fig 2G and 2J and S1 Fig). In the stria vascularis, the signal of the α6(IV) chain colocalized at the outer side of α2(IV) chain-containing BMs, which surround the endothelium and pericytes (Fig 2H and 2K).

### Differential composition of collagen IV networks in the mouse cochlea

**Fig 3:**
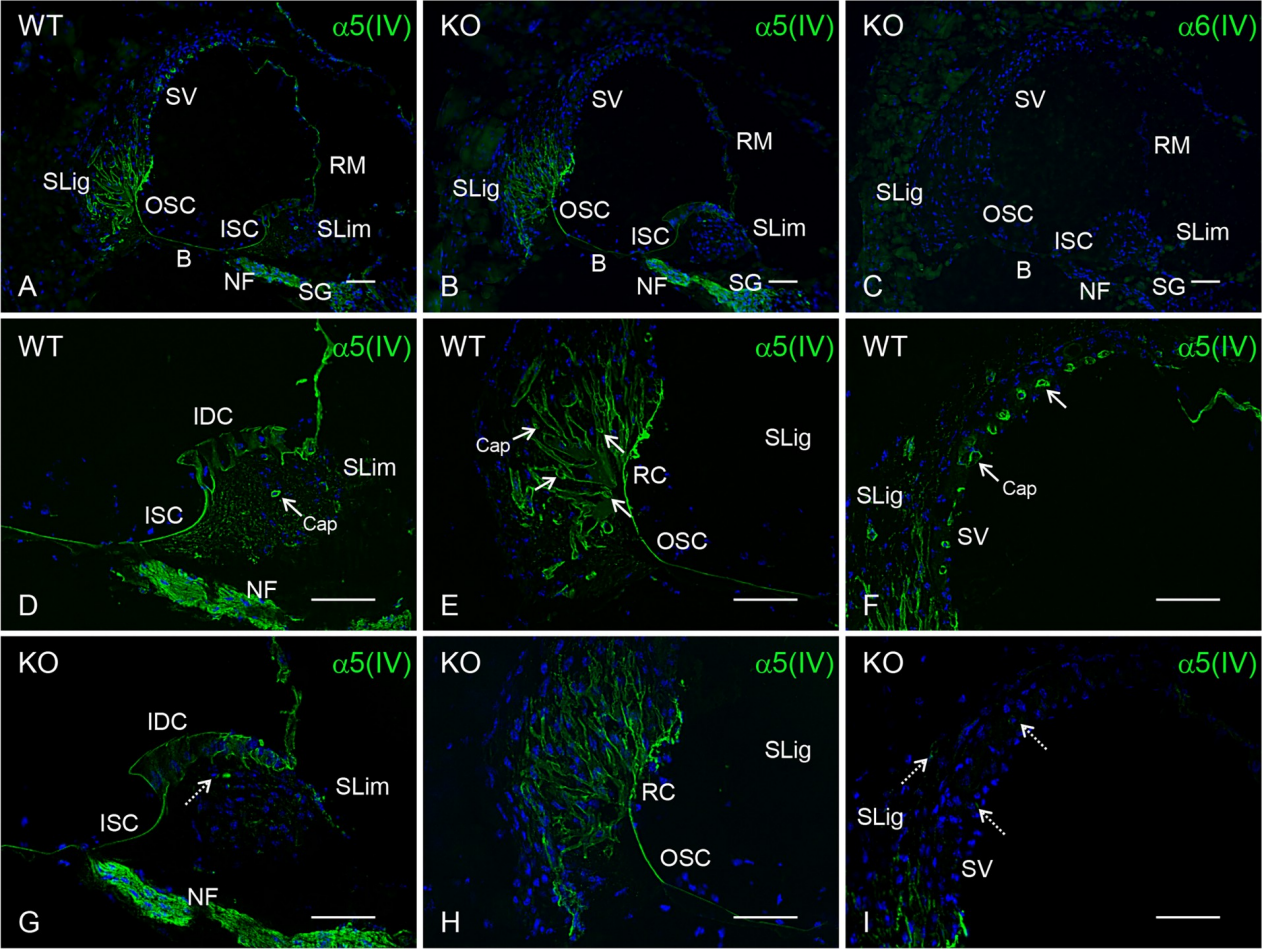
Distribution of collagen α5(IV) chain in the cochlea of WT and *Col4a6* KO mice. (A, D–F) The collagen α5(IV) chain located in the subepithelial BMs, perivascular BMs, and perineural BMs in WT mice (green). (B, G–I) In *Col4a6* KO mice, the α5(IV) chain is widely distributed (green). However, weak signals were detected in the perivascular BMs of the spiral limbus (SLim), spiral ligament (SLig), and stria vascularis (SV) (dashed arrows). (C) No immunopositive signal of the collagen α6(IV) chain was present in *Col4a6* KO mouse cochlea (green). Nuclei were counterstained with Hoechst 33258 (blue). B, basilar membrane; RM, Reissner’s membrane; NF, neural fasciculus; SG, spiral ganglion; IDC, interdental cells; ISC, inner sulcus cells; OSC, outer sulcus cells; RC, root cells; Cap, capillary. Scale bars = 50 μm.

We performed immunohistochemistry to investigate the molecular composition of cochlear BMs using *Col4a6* KO and WT mice. In WT mice, collagen α5(IV) chain was distributed in the subepithelial BMs of interdental cells, inner sulcus cells, basilar membrane, outer sulcus cells, root cells, and Reissner’s membranes; perivascular BMs in the spiral limbus, spiral ligament, and stria vascularis; and the perineural BMs of the neural fasciculus and spiral ganglion (Fig 3A and 3D–3F). Based on the results for the collagen α6(IV) chain, α5α6α5 was suggested to be present in all subepithelial BMs and perivascular BMs in the mouse cochlea (Figs 2 and 3).

Notably, the collagen α5(IV) chain was observed in the subepithelial BMs and perineural BMs, however, it was scarcely detected in the perivascular BMs of *Col4a6* KO mice (Fig 3B and 3G–3I). Moreover, images with longer exposure showed weak α5(IV) chain immunostaining in perivascular BMs from *Col4a6* KO mice (S2 Fig). Similarly, α3(IV) and α4(IV) chains were weakly immunopositive in the perivascular BMs (S2 Fig). Since collagen IV protomer assembly requires three α(IV) chains, the lack of any one of these chains would result in a failure to assemble a triple-helical form by the remaining two chains [8]. Collectively, our results suggest that α1α2α1, α3α4α5, and α5α6α5 in the subepithelial and perivascular BMs, and α1α2α1 and α3α4α5 in the perineural BMs contribute to the assembly of the collagen IV network. α5α6α5, in addition to α1α2α1, is a quantitatively major component of the collagen IV network in the perivascular BMs. Table 1 presents the tissue distribution of collagen IV in mouse cochlea.

**Table 1:** Qualitative assessment using immunohistochemistry: Distribution of collagen α1(IV), α2(IV), α5(IV), and α6(IV) chains and the predicted molecular composition of the collagen IV network in mouse cochlea.

Classification¤	Location of BMs¤	α(IV) ¤				Predicted heterotrimer¤		
		α1^a^¤	α2^a^¤	α5^a^¤	α6^b^¤	α1α2α1^a^¤	α3α4α5^a^¤	α5α6α5^b^¤
**Subepithelial BMs**¤	**Interdental cells**¤	+¤	+¤	+¤	+¤	+¤	+¤	+¤
	**Inner sulcus cells**¤	+¤	+¤	+¤	+¤	+¤	+¤	+¤
	**Basilar membrane**¤	+¤	+¤	+¤	+¤	+¤	+¤	+¤
	**Outer sulcus cells**¤	+¤	+¤	+¤	+¤	+¤	+¤	+¤
	**Root cells**¤	+¤	+¤	+¤	+¤	+¤	+¤	+¤
	**Reissner’s membrane**¤	+¤	+¤	+¤	+¤	+¤	+¤	+¤
**Perivascular BMs**¤	**Spiral limbus**¤	+¤	+¤	+¤	+¤	+¤	+^**b**^¤	+¤
	**Spiral ligament**¤	+¤	+¤	+¤	+¤	+¤	+^**b**^¤	+¤
	**Stria vascularis**¤	+¤	+¤	+¤	+¤	+¤	+^**b**^¤	+¤
**Perineural BMs**¤	**Neural fasciculus**¤	+¤	+¤	+¤	-¤	+¤	+¤	-¤
	**Spiral ganglion**¤	+¤	+¤	+¤	-¤	+¤	+¤	-¤

BM, Basement membrane; +, positive; -, negative.

^a^ Its presence was previously identified [28, 30].

^b^ Data shown for the first time in the current study. α3α4α5(IV) protomer in the perivascular BMs was a quantitatively minor component of the collagen IV network.

### Assessment of hearing threshold by click-evoked ABR

**Fig 4:**
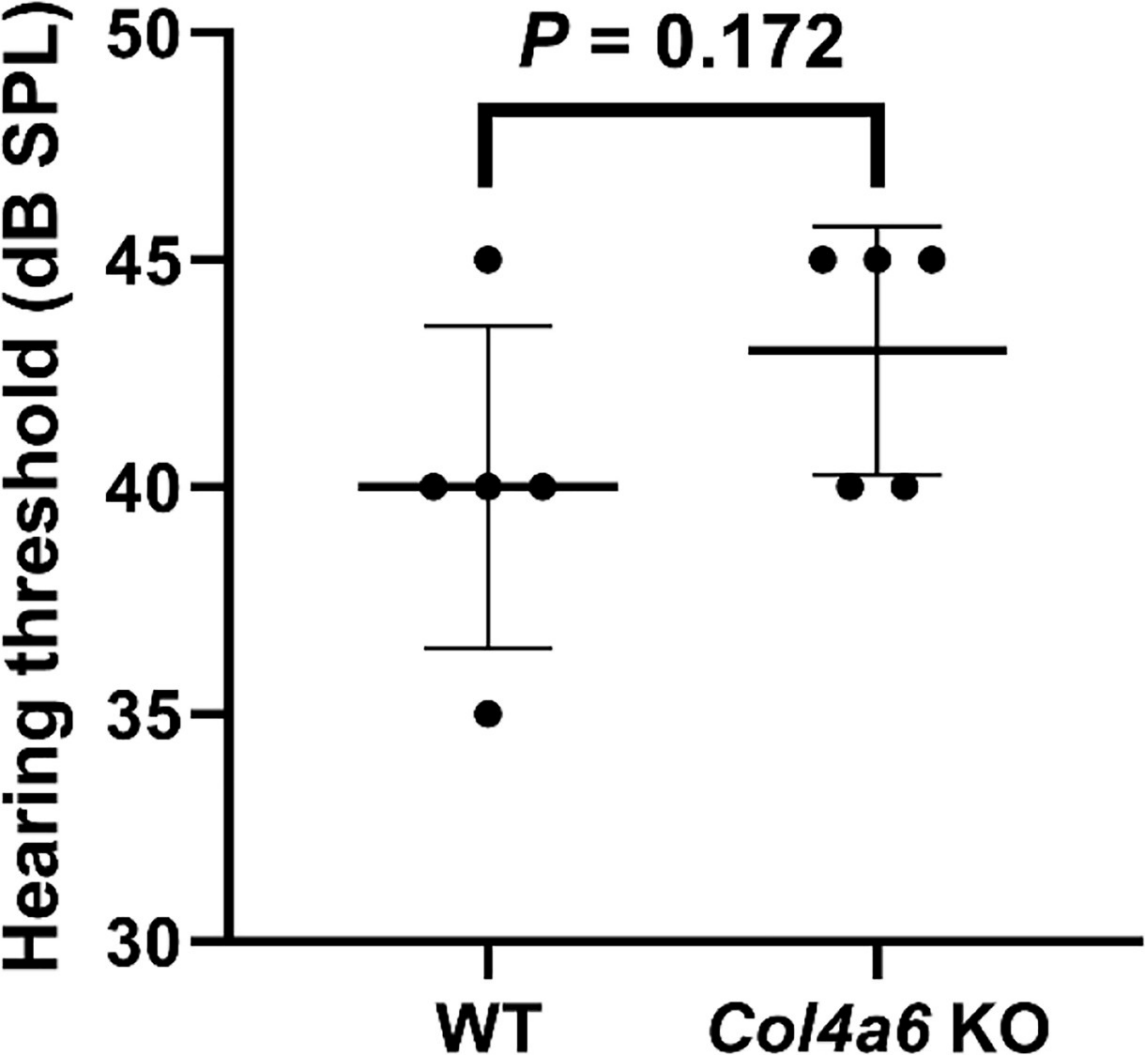
Hearing threshold assessed by click-evoked auditory brainstem responses in WT and *Col4a6* KO mice. No significant difference observed between WT mice (mean 40 ± 3.5 dB SPL, n = 5) and *Col4a6* KO mice (mean 43 ± 2.7 dB SPL, n = 5). *P* = 0.172 by Student’s unpaired *t*-tests. The values are presented as mean ± SD.

To assess the hearing function in *Col4a6* KO mice, we compared the click-ABR thresholds of WT mice to those of our *Col4a6* KO mice (8-week-old male WT mice, C57BL/6J background). The average threshold of the WT group and *Col4a6* KO group were 40 ± 3.5 dB SPL and 43 ± 2.7 dB SPL, respectively (means ± SD, n = 5). The hearing threshold was not significantly different between WT and *Col4a6* KO mice (*P* = 0.172, Fig 4).

### Micro-CT analysis

**Fig 5:**
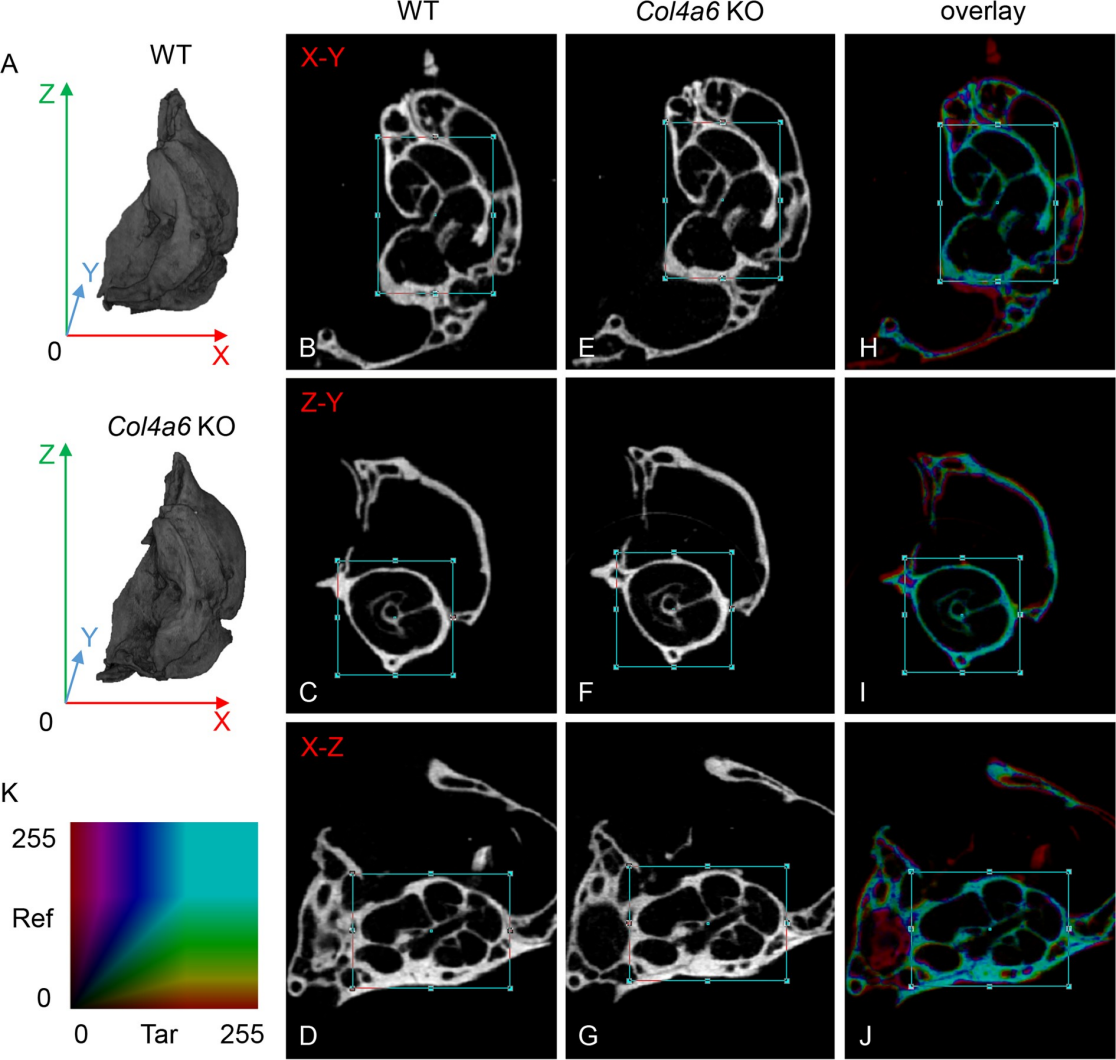
Comparison of the cochlea bone structure of WT and *Col4a6* KO mice by micro-CT analysis. (A) Representative three-dimensional images of the petrous portion of the left temporal bone in WT and *Col4a6* KO mice. The top shows the apex of petrous in the superior view. (B–G) The three orthogonal plane images were automatically obtained as a best-matched plane from the *Col4a6* KO data set compared to the images manually selected from the cochlea structure of the WT mice data set. (H–J) The overlay images demonstrated that no differences in the size or shape are apparent in the cochlear bone between WT and *Col4a6* KO mice. Blue squares represent the Volume of Interest. (K) Light blue color indicates a highly matched degree based on the color map.

Patients with hearing loss caused by the X-linked hereditary mutation of *COL4A6* were reported to have malformed cochlea, as determined using high-resolution CT [22]. Therefore, to investigate the structural abnormalities in *Col4a6* KO mouse cochlea, we performed a micro-CT analysis. No differences were observed in the three-dimensional images of the petrous portion of the left temporal bone between the two groups of mice (Fig 5A). Moreover, three kinds of orthogonal 2-D cross-sections, including the transverse plane (X-Y), sagittal plane (Z-Y), and coronal plane (X-Z), were compared using DataViewer software. No abnormalities were found in the *Col4a6* KO cochlea structure (Fig 5B–5G). Furthermore, the overlay images based on the color map showed no differences in the size or shape of the cochlea between WT and *Col4a6* KO mice (Fig 5H–5K). Taken together, our results demonstrate that *Col4a6* KO mice have no gross malformation of the osseous labyrinth.

### Histological analysis

**Fig 6:**
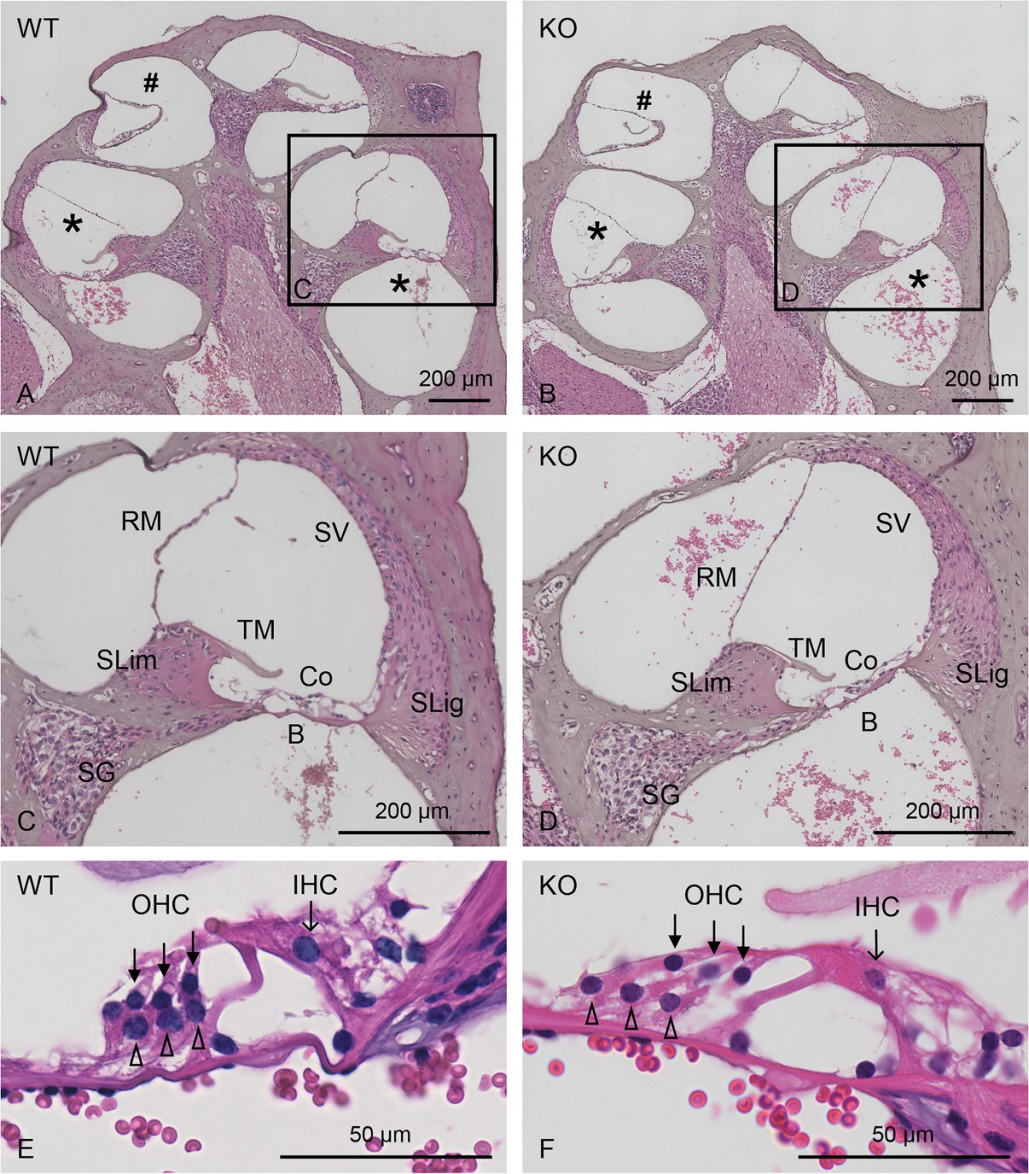
Representative images of HE-stained midmodiolar cochlear sections. Histological comparison between WT (A, C, E) and *Col4a6* KO (B, D, F) mice. (C-F) Images with higher magnification of cochlear basal turn are shown. No differences are observed between WT and *Col4a6* KO mice. ⁎, basal turn; #, apical turn; SG, spiral ganglion; SLim, spiral limbus; TM, tectorial membrane; B, Basilar membrane; Co, organ of Corti; SLig, spiral ligament; SV, stria vascularis; RM, Reissner’s membrane; closed arrows, OHC (outer hair cells); arrows, IHC (inner hair cells); open arrowheads, Dieter’s cells.

To achieve a more detailed analysis of cochlear formation, both WT and *Col4a6* KO (8-week-old) mouse cochlea were compared histologically using HE-stained midmodiolar sections (Fig 6). *Col4a6* KO mice exhibited regular morphological structures through the basal to apical turn, as those observed in WT mice, comprising spiral limbus followed by inner sulcus, spiral ganglion, and neural fasciculus extended to the organ of Corti, which adheres to the basilar membrane (Fig 6A and 6B). Moreover, the tectorial membrane, spiral ligaments, stria vascularis, and Reissner’s membrane were similar in structure between the two groups (Fig 6C and 6D). The outer and inner hair cell rows, accompanied by Dieter’s cells, also showed regular morphology in *Col4a6* KO and WT mice (Fig 6E and 6F). Hence, no significant differences in any structure were detected in *Col4a6* KO mice compared to WT mice (n = 3).

### No change in the distribution of major BM components

**Fig 7:**
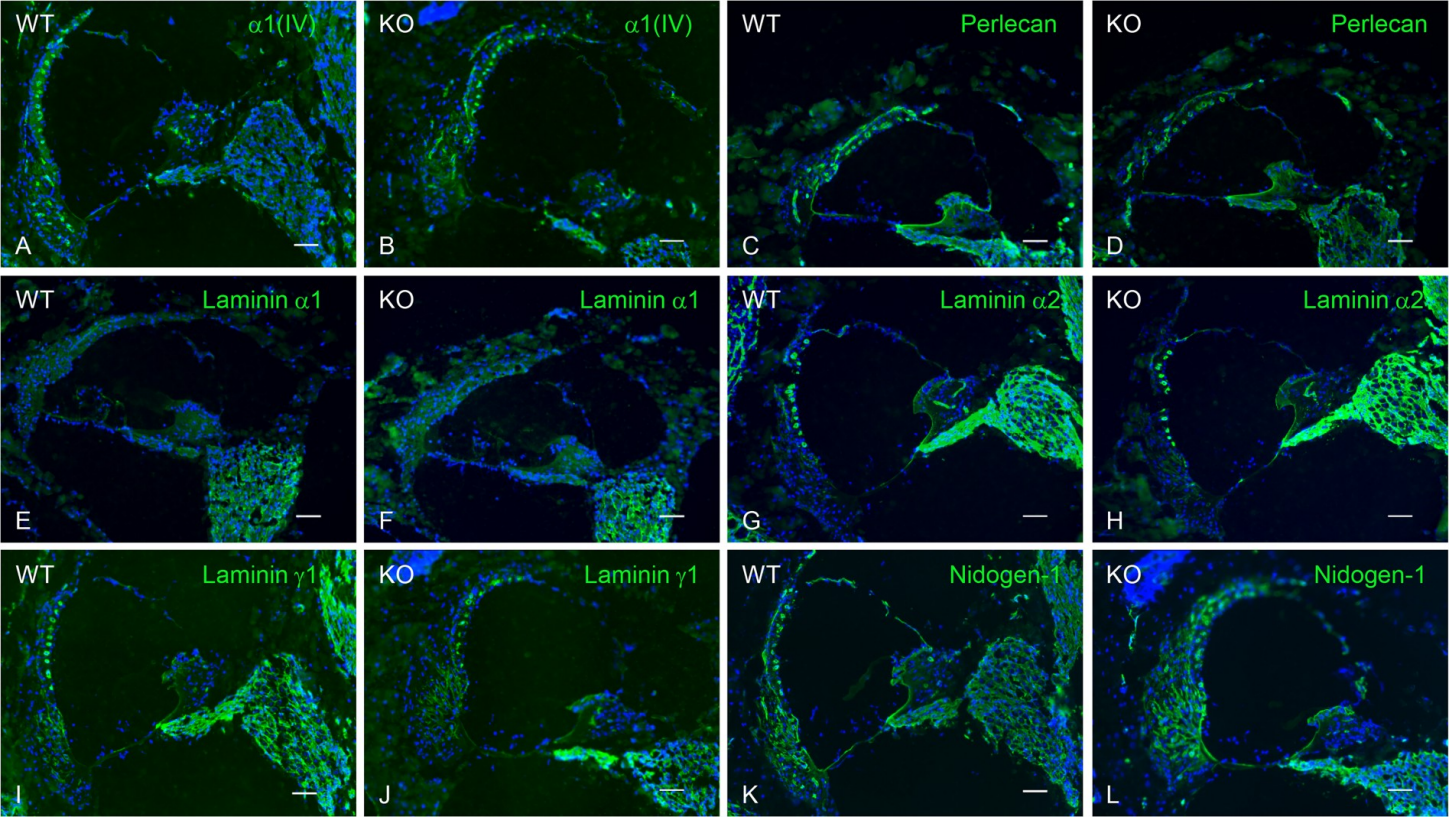
Distribution of major basement membrane components in the cochlea of WT and *Col4a6* KO mice. Cryosections were immunostained by antibodies against collagen α1(IV) chain (A, B), perlecan (C, D), laminin α1 (E, F), laminin α2 (G, H), laminin γ1 (I, J), and nidogen-1 (K, L). Nuclei were counterstained with Hoechst 33258 (blue). No differences are apparent between WT and *Col4a6* KO mice. Scale bars = 50 μm.

To examine the changes in the expression of the other major BM components, we performed immunohistochemistry. No apparent differences in the staining pattern of collagen α1(IV) chain, perlecan, laminin α1, laminin α2, laminin γ1, or nidogen-1 were detected in the cochlea of *Col4a6* KO and WT mice (Fig 7).

## Discussion

In this study, we characterized the localization of the collagen α6(IV) chain in mouse cochlea and demonstrated that the loss of collagen α6(IV) chain expression showed normal click ABR thresholds and normal cochlear formation, which differs from humans with the *COL4A6* missense mutation c.1771G>A, p.Gly591Ser.

Our results showed that the collagen α6(IV) chain is expressed in both subepithelial BMs in the interdental cells, inner sulcus cells, basilar membrane, outer sulcus cells, root cells, and Reissner’s membrane, as well as the perivascular BMs in the spiral limbus, spiral ligament, and stria vascularis. A previous study reported that the α6(IV) chain exists intracellularly in a subgroup of ganglia cells, while our results, using monoclonal antibody B66, did not detect signals in these cells [22]. B66 has been established as a monoclonal antibody that specifically reacts with the α6(IV) chain of human, bovine, and mice [9, 11, 32]. We reconfirmed that B66 had no immunopositive signals in the tissue derived from *Col4a6* KO mice (Fig 3C). In addition, B66 showed clear immunopositive signals corresponding to the presence of BMs in the mouse cochlea. On the other hand, the distribution of α3α4α5 in the cochlea of WT and *Col4a3* KO mice by immunohistochemistry using antibodies against collagen α3(IV), α4(IV), and α5(IV) chains has been shown in previous reports [28, 30]. In this study, we presented immunohistochemistry data using specific antibodies against each collagen α(IV) chain; hence, comparison of the distribution of collagen α1(IV) to α6(IV) chains represented the tissue-specific collagen IV network of the mouse cochlea (Table 1). In particular, we demonstrated the presence of α1α2α1, α3α4α5, and α5α6α5 in subepithelial BMs, α1α2α1, α3α4α5, and α5α6α5 in perivascular BMs, and α1α2α1 and α3α4α5 in perineural BMs.

The stria vascularis is responsible for supporting the endocochlear potential and high potassium content of the endolymph in the cochlear duct, which is necessary for sensory hair cell conduction [33]. The structure comprises marginal cells, intermediate cells, basal cells, and a dense capillary network. The capillaries and intermediate cells are located between the marginal cell layer and the basal cell layer. Moreover, the perivascular BMs surround the endothelial cells and pericytes in the capillary network, which is contacted by the marginal cells and intermediate cells [34]. BMs regulate various cell functions, such as stable anchoring, proliferation, and migration, primarily mediated by integrin, a major cell surface receptor for the extracellular matrix. In addition, BMs serve as a filter for electrically charged molecules [5]. In fact, perivascular BMs may participate in the blood-labyrinth barrier, which is essential for preventing the influx of harmful substances into the intrastrial space, similar to the blood-brain barrier [35, 36]. Moreover, double-layered BMs are detected in the stria vascularis, which are derived from endothelial cells, pericytes, and marginal and intermediate epithelial cells [34, 37]. Hearing impairment that is age-related, drug-induced, or results from acute injury or genetic disease, such as Alport syndrome (AS), is associated with changes in the structure and components of the perivascular BMs in the stria vascularis [28, 38–41]. Interestingly, the collagen α6(IV) chain was detected in the outer layer of BMs surrounding the capillary network of the stria vascularis, suggesting that the α6(IV) chain-containing BMs may functionally be related to marginal and intermediate epithelial cells.

Recently, a Hungarian family was reported to have congenital X-linked nonsyndromic hearing loss caused by a mutation in *COL4A6* (c.1771G>A, p.Gly591Ser), which was accompanied by cochlear malformation. All male members of this family with the *COL4A6* mutation showed severe-to-profound hearing loss at all frequencies tested (0.125 to 8 kHz), which began during early childhood. Bioinformatic analysis showed that this mutation produces an atypical large side-chain in the interchain of the heterotrimer, which reduces the stability of the triple-helix and likely triggers disarrangement of the quaternary structure [22]. However, the current study demonstrates that the click ABR thresholds, histology, and micro-CT scanning images of *Col4a6* KO mice did not differ compared to those of WT mice. We also examined the possibility that other BM components compensated for the deleterious effect induced by the absence of the collagen α6(IV) chain in the cochlea. However, immunohistochemistry staining showed that the expression of major BM components did not differ between *Col4a6* KO and WT mice. Taken together, our results suggest that a null-type mutation of *Col4a6* in mice had no deleterious impact on cochlear formation or on click ABR thresholds. Our characterization of the *Col4a6* KO mice differs dramatically from the phenotype observed in humans with the *COL4A6* missense mutation c.1771G>A, p.Gly591Ser.

Collagens constitute a superfamily of extracellular matrix proteins that function primarily as structural proteins. Several collagen genes (*COL1A1*, *COL1A2*, *COL2A1*, *COL4A3*, *COL4A4*, *COL4A5*, *COL11A1*, *and COL11A2*) are associated with hereditary syndromic hearing loss. Mutations in *COL11A1* and *COL11A2* have been shown to cause nonsyndromic hereditary hearing loss [42, 43]. Osteogenesis imperfecta (OI) is commonly caused by autosomal dominant mutations in the genes encoding collagen I and is characterized by growth deficiency and vulnerability to fractures from minimal trauma [44]. The molecular defect in type I OI, the mildest form, is a null *COL1A1* allele caused by frameshifts or a premature stop codon, resulting in reduced synthesis of structurally normal collagen, whereas types II–IV OI, the more severe forms, are caused by defects in the collagen I fibril structure, most commonly glycine substitutions (80%). The mutated collagen α(I) chain delays the folding of the heterotrimer and results in over-post-translational modification. Misfolded heterotrimers then induce the unfolded protein response (UPR) in the endoplasmic reticulum, associated with a pathogenic defect in the differentiation and maturation of osteoblasts. In cases where abnormal procollagen can escape from the intracellular degradation pathway and are secreted, the collagen fiber with a misfolded heterotrimer has lower integrity and affects the mechanical strength and various cell-matrix interactions [45]. Moreover, AS is a hereditary disorder characterized by progressive renal failure, sensorineural hearing loss, and ocular defects caused by mutations in *COL4A3*, *COL4A4*, and *COL4A5*. To date, over 500 mutations have been identified, including point mutations, deletions, and insertions of a large or small nucleotide sequence [46–49]. Most mutations result in failure of assembly and/or secretion of collagen α3α4α5(IV) heterotrimers, and consequently, all three α chains are missing from the tissue. While collagen α3α4α5(IV) heterotrimers with subtle missense mutations can be assembled, secreted, and form the network in some cases; however, it still results in milder disease severity in patients due to the functionally impaired BMs [48]. For instance, Naito *et al*. showed sparse or normal immunostained signals of the collagen α5(IV) chains harboring the glycine substituted missense mutation in the glomerular BM of patients [50]. Of note, a missense mutation, *COL4A3*-G1334E, leads to collagen α3α4α5(IV) heterotrimer misfolding, which causes podocyte UPR [51]. Besides, chemical chaperones have recently been reported to have therapeutic potential for AS by normalizing the mutated collagen α3α4α5(IV) heterotrimer [52].

Collectively, the missense mutation of *COL4A6* (c.1771G>A, p.Gly591Ser) may affect hearing impairment as a consequence of the aberrant function associated with the collagen α6(IV) chain due to a misfolded collagen heterotrimer (Fig 1). Although the present study was not designed to show the direct effect of the mutated collagen α6(IV) chain on hearing function, our findings might provide a basis for developing advanced applications of a *Col4a6* knock-in mouse model carrying the missense mutation. Further studies are necessary to elucidate the pathogenesis of hereditary hearing loss and cochlear malformation associated with *COL4A6* missense mutations.

The primary limitation of this study is that the analysis of hearing levels in mice was exclusively based on the click-ABR method, which does not provide information on the frequency-specific hearing levels. Hearing assessment in our future studies will include ABR using pure tones to analyze hearing levels in low, middle, and high frequencies. Moreover, although the present study detected no histological differences in the organ of Corti between WT and *Col4a6* KO mice, via light microscopy, *Col4a6* KO mice may have BM functional impairment of the organ of Corti along the tonotopic axis of the apical, middle, and basal cochlear turns, which are involved in sound transduction at each frequency. In addition, startle response testing will provide insight into behavioral responses to brief, intense sound and otoacoustic emissions testing will further evaluate the function of outer hair cells along the tonotopic axis of the cochlea.

## Supporting information

S1 Fig Distribution of collagen α1(IV) and α2(IV) chains in the mouse cochlea. Cryosections were immunostained with antibodies against α1(IV) (A–F: green) and α2(IV) chains (G–L: green). Nuclei were counterstained with Hoechst 33258 (blue). SLim, spiral limbus; NF, neural fasciculus; SG, spiral ganglion; B, basilar membrane; SLig, spiral ligament; SV, stria vascularis; RM, Reissner’s membrane. Scale bars = 50 μm.
(TIF)

S2 Fig Distribution of collagen α3(IV), α4(IV), and α5(IV) chains in the perivascular basement membranes of WT and Col4a6 KO mice. Cryosections were immunostained with antibodies against α3(IV) (A: green), α4(IV) (B: green), α5(IV) chains (C–F: green), and CD31 (D–F: red). The results suggest that α3α4α5(IV) heterotrimer is present in the perivascular BMs of the mouse cochlea. (A–C) The subepithelial BMs in the spiral ligament (SLig) were used as positive controls for each antibody in the inset. (G–I) The negative control was established by immunostaining with only secondary antibody; no specific reaction was observed. Nuclei were counterstained with Hoechst 33258 (blue). Arrows, immunopositive perivascular BMs in the spiral ligament (SLig); open arrowheads, immunopositive perivascular BMs in the stria vascularis (SV); SLim, spiral limbus. Scale bars = 50 μm. Scale bars in inset = 20 μm.
(TIF)
