# Supplementary figures and images for: Lack of collagen α6(IV) chain in mice does not cause severe-to-profound hearing loss or cochlear malformation, a distinct phenotype from nonsyndromic hearing loss with COL4A6 missense mutation
Source: PLoS One. 2021 Apr 13;16(4):e0249909. doi: 10.1371/journal.pone.0249909 (PMC8043391; doi:10.1371/journal.pone.0249909)

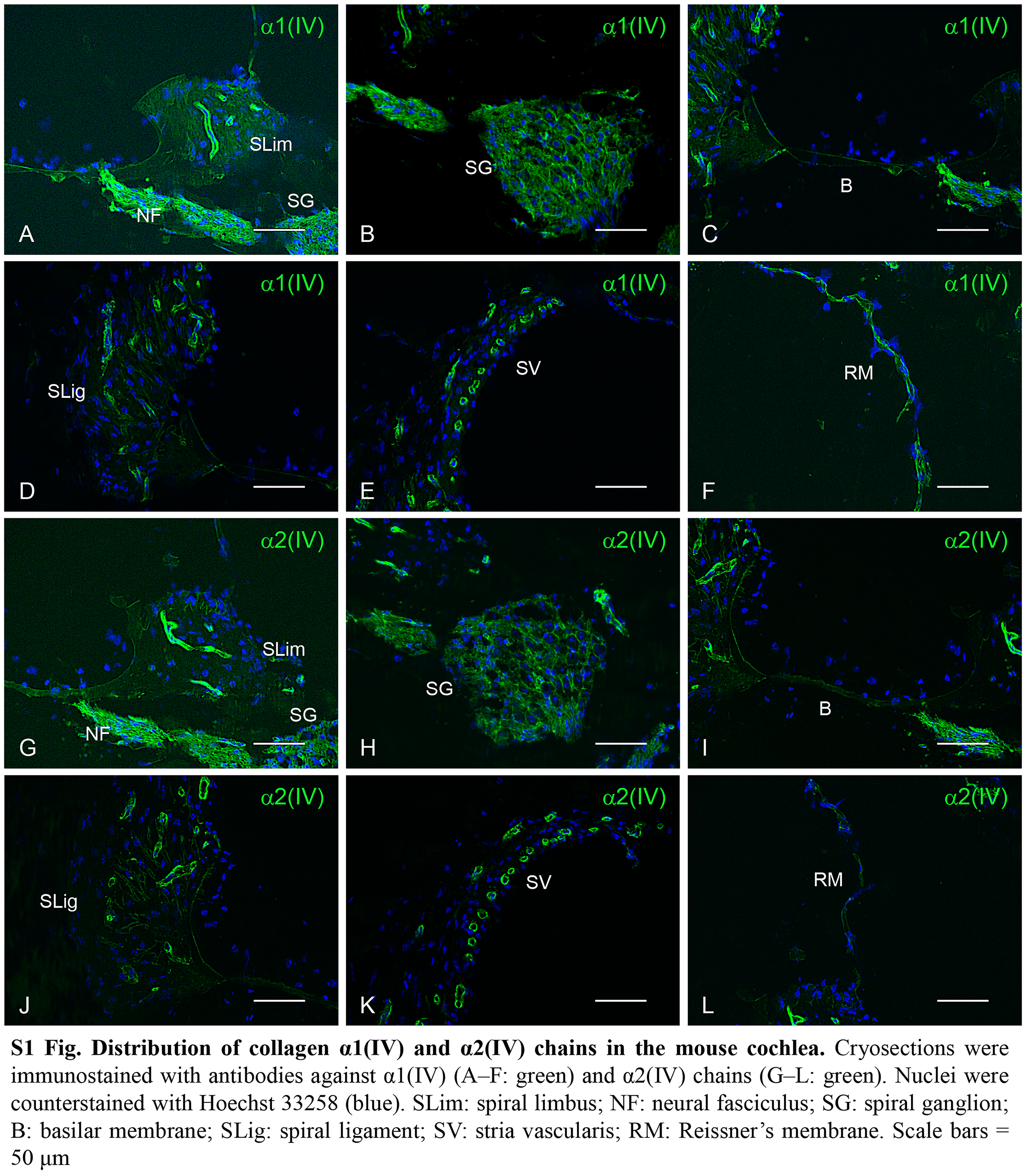

Supplement: S1 Fig — Cryosections were immunostained with antibodies against α1(IV) (A–F: green) and α2(IV) chains (G–L: green). Nuclei were counterstained with Hoechst 33258 (blue). SLim, spiral limbus; NF, neural fasciculus; SG, spiral ganglion; B, basilar membrane; SLig, spiral ligament; SV, stria vascularis; RM, Reissner’s membrane. Scale bars = 50 μm. (TIF) [file pone.0249909.s001.tif]

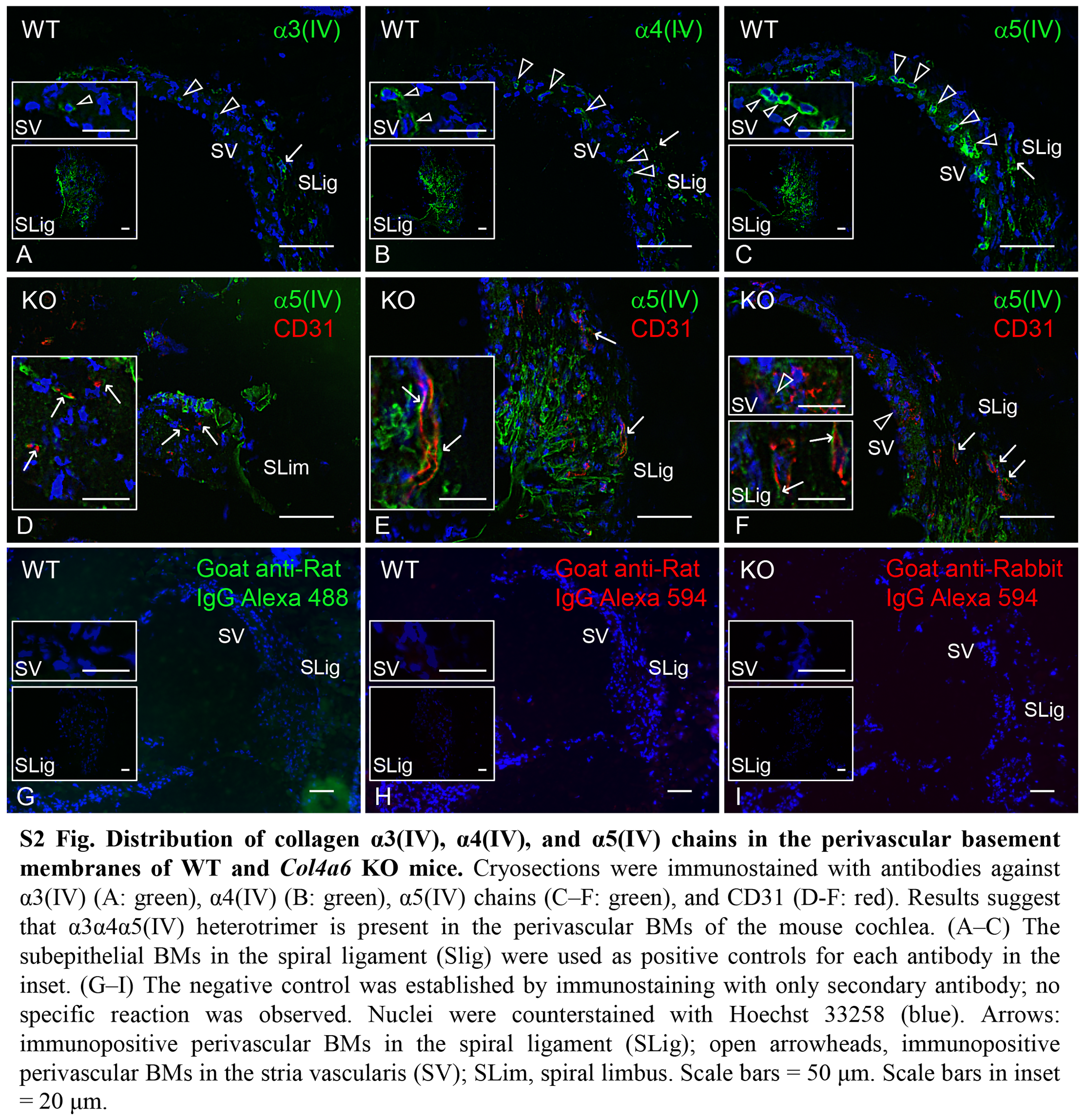

Supplement: S2 Fig — Cryosections were immunostained with antibodies against α3(IV) (A: green), α4(IV) (B: green), α5(IV) chains (C–F: green), and CD31 (D–F: red). The results suggest that α3α4α5(IV) heterotrimer is present in the perivascular BMs of the mouse cochlea. (A–C) The subepithelial BMs in the spiral ligament (SLig) were used as positive controls for each antibody in the inset. (G–I) The negative control was established by immunostaining with only secondary antibody; no specific reaction was observed. Nuclei were counterstained with Hoechst 33258 (blue). Arrows, immunopositive perivascular BMs in the spiral ligament (SLig); open arrowheads, immunopositive perivascular BMs in the stria vascularis (SV); SLim, spiral limbus. Scale bars = 50 μm. Scale bars in inset = 20 μm. (TIF) [file pone.0249909.s002.tif]
